# When ethnic minorities hit the headlines: The longitudinal associations between news features and adolescents' ethnic prejudice

**DOI:** 10.1111/jora.13013

**Published:** 2024-09-03

**Authors:** Beatrice Bobba, Adele Miniati, Elisabetta Crocetti

**Affiliations:** ^1^ Department of Psychology Alma Mater Studiorum University of Bologna Bologna Italy

**Keywords:** adolescence, ethnic prejudice, longitudinal, media, newspaper

## Abstract

Ethnic prejudice poses great challenges to adolescents' adjustment to multicultural societies. However, little is known about the role of the media in influencing attitudes in adolescence. Combining information environment and ecological development theories, the current study examined the longitudinal associations between the quantity, valence (i.e., neutral, positive, and negative), and target (i.e., migrant, refugee, and foreigner) of the news about ethnic minority groups and youth's affective and cognitive prejudice. In total, 962 adolescents (*M*_age_ = 15.67, 48.13% females) completed questionnaires at two time points, and news data were gathered from a national newspaper. While news quantity did not matter, positive and negative news were respectively associated with reduced and increased levels of cognitive, but not affective, prejudice. Nuanced associations emerged when accounting for the news target. Results were replicated regardless of adolescents' direct consumption of newspapers. These findings highlight the role of the information environment and suggest the need to account for it in planning interventions.

Over the past decades, migration‐related issues have been at the core of social and political discussions as a consequence of increasing in‐flows in both Europe and the United States (Grande et al., 2019; van der Brug et al., 2015). Along this line, electoral campaigns in several countries have revolved around issues of migration and border control and political stances have been taken against ethnic minority groups within a given context. The media contribute to these debates by leaning on specific representations of migrants and ethnic minorities and conveying threatening depictions of ethnic diversity (Schaub & Morisi, 2020). For instance, the more media report negative portrayals (e.g., as criminals or economic threats) of ethnic minority individuals, the higher the prejudice against and the concerns about these groups get (e.g., Boomgaarden & Vliegenthart, 2009; Conzo et al., 2021; Fuochi et al., 2020). However, most theoretical and empirical research has focused on the quantity and valence of the news about ethnic minorities and how being *directly* exposed to them might influence individual attitudes about members of the ethnic minority group. Conversely, less is known about other features (e.g., target of the news) of the media environment and how they can *indirectly* contribute to youth's perceptions of ethnic minorities.

Building upon these premises, this study sought to test whether, in line with the information environment theoretical approach (Boomgaarden & Vliegenthart, 2009; Weimann & Brosius, 1994), multiple features (i.e., quantity, valence, and target) of the news about ethnic minorities can be indirectly related to individuals' feelings and thoughts about others, by creating a general ecological macro‐context where salient issues can shape how ethnic diversity is approached. This might be especially relevant during adolescence, when youth face the crucial task of consolidating personal beliefs, attitudes, and orientations toward the self (Crocetti et al., 2023) and others (Knifsend & Juvonen, 2014; Meeus, 2019). These developmental goals are achieved as a result of important advancements in social (e.g., empathic competences; Van der Graaff et al., 2014) and cognitive (e.g., more sophisticated reasoning; Kuhn, 2009) abilities coupled with the influences of the multiple contexts (e.g., family, school, and society) within which youth are embedded (Bronfenbrenner, 1992, 2005). Therefore, the current study aims to examine whether (and how) the quantity, valence (i.e., neutral, positive, and negative), and target (i.e., migrant, refugee, and foreigner) of the news about ethnic minority groups are linked to changes in adolescents' ethnic prejudice and whether these effects are maintained regardless of youth's actual direct consumption of newspaper.

## Ethnic prejudice: A multidimensional and complex phenomenon

Ethnic prejudice can be defined as a form of antipathy toward a group of individuals (i.e., minority or outgroup) because of their different ethnic and cultural background (Allport, 1954). It is a multifaceted and complex phenomenon, encompassing affective, cognitive, and behavioral dimensions (Brown, 2011). The affective component refers to the domain of emotions, such as liking or disliking people belonging to an ethnic minority. Conversely, the cognitive component refers to negative beliefs, attitudes, and stereotypes attributed to outgroup members. Together, the affective and cognitive dimensions form the basis for the behavioral expression of ethnic prejudice (Cuddy et al., 2007), which includes, among others, avoidance, discrimination, or marginalization of ethnic minority individuals. Despite displaying overall stability over the course of adolescence (for meta‐analyses, see Crocetti et al., 2021; Raabe & Beelmann, 2011), affective and cognitive dimensions of prejudice might be differently influenced by individual and socio‐contextual factors (e.g., Beelmann & Heinemann, 2014).

Along this line, prior research has extensively examined the interplay between the proximal contexts of development (e.g., family and school) and adolescents' ethnic prejudice levels (e.g., Bobba, Branje, & Crocetti, 2024). However, these contexts are themselves embedded in and influenced by distal macro‐contextual factors (e.g., culture, media) and conditions (e.g., historical events) that can ultimately shape how youth think and feel about ethnic diversity (Bronfenbrenner, 1992, 2005). Specifically, the public discourses and the news conveyed by the media might define a general macro‐context within which intergroup conflicts, threat perceptions, and social inequalities are maintained and even reinforced (Dovidio et al., 2010). Therefore, it is crucial to understand how specific aspects of the news environment can be intertwined with adolescents' thoughts and feelings about ethnic minority groups in current multicultural societies (Bagci & Rutland, 2019).

## Media news and ethnic prejudice

Media can play an important role in shifting individual opinions about a wide range of societal issues, including migration, ethnic diversity, and intergroup experiences (for a review, see Mastro, 2009). The role of media has been mainly examined within social (i.e., intergroup contact; Allport, 1954) and communication (i.e., media priming and valence framing theories; Boomgaarden, 2007) approaches. Both perspectives consider the direct exposure to media representations of ethnic minority groups as a source of information and priming (e.g., Dixon & Azocar, 2007) about the members of such groups, which can influence later intergroup evaluations and personal attitudes (Allport, 1954; Joyce & Harwood, 2014).

Beyond the direct influences exerted by consuming the media, the news content can also be conceptualized as a contextual and macro‐level variable. Along this line, the information environment approach (Boomgaarden & Vliegenthart, 2009; Weimann & Brosius, 1994) contends that the news can influence individual perceptions and beliefs also indirectly, by defining an ecological macro‐context within which certain aspects of ethnic minorities are emphasized and others neglected. Due to the overlap in news visibility across different media outlets, the information environment can also reach those who are not directly exposed to it because it sets a dominant perspective that is also transmitted via interpersonal communication. In turn, these macro‐contextual representations can contribute to shifts in public opinion and individual prejudice levels (Jerit et al., 2006). Building upon these premises, the current study examines whether different features (i.e., quantity, valence, and target) of the news about ethnic minorities reported in a national newspaper can contribute to changes in adolescents' affective and cognitive prejudice, regardless of youth's actual direct consumption of this media outlet.

### How news features are linked to ethnic prejudice: The role of quantity and valence

Most research conducted within either the social (Allport, 1954), the communication (Boomgaarden, 2007), or the information environment (Boomgaarden & Vliegenthart, 2009; Weimann & Brosius, 1994) approaches has emphasized the role of two features of the news, mainly the quantity and valence of information about a given group (Eberl & Galyga, 2021). On the one hand, the *quantity* of news about migration to which individuals were directly exposed has been linked to heightened intergroup anxiety (Visintin et al., 2017, Study 1), negative attitudes toward migrants (Fuochi et al., 2020, Study 1), and stereotypical representations of African American in the U.S. context (e.g., Dixon, 2008). Similarly, concerns about migration in the general population were found to increase when the information environment focused more on migration‐related issues (Czymara & Dochow, 2018). On the other hand, the *valence* (i.e., neutral, positive, and negative) of media representations of ethnic minorities can skew individual attitudes and beliefs about them in one direction or the other. For instance, positive messages (e.g., support for the reduction of intergroup conflict, positive portrayals of minority; e.g., Mastro & Tukachinsky, 2011; Paluck, 2009; Schemer, 2012) and negative stereotypes (e.g., minority members as criminals, violent, or threatening; Conzo et al., 2021; Fuochi et al., 2020; Schemer, 2012) were found to foster better and worse attitudes toward ethnic diversity, respectively.

Interestingly, only limited research has examined whether the quantity and valence of the news could be differently associated with multiple dimensions of ethnic prejudice. A recent experimental study has highlighted that exposure to valenced news about ethnic minorities led to changes in adults' affective prejudice. In contrast, other subtler linguistic components of the news were mostly linked to its behavioral facet. Specifically, nouns (e.g., «*a Jew*») have a stronger inductive potential compared to adjectives (e.g., «*a Jewish person*») and activate the stereotypes attached to a given label, supporting category‐congruent inferences (Carnaghi et al., 2008, p. 841; see also, Graf et al., 2013). Relatedly, using nouns to identify ethnic minorities was consistently linked to increased desire for social distance from them, regardless of the news valence (Graf et al., 2020). These findings suggest the need to account for the multifaceted nature of ethnic prejudice. They also underline the importance of considering additional features of the news to comprehensively understand whether and how media content is related to youth's feelings and attitudes against ethnic minority groups.

### Ethnic minority groups defined in the media: The role of news target

Consistent evidence shows that newspapers tend to provide a greater amount of negative information about ethnic minorities across different contexts (e.g., Dragojevic et al., 2017; Visintin et al., 2017). However, this is not the only way in which media can reflect and perpetuate societal discrimination against diverse others. Specifically, beyond the effect of news quantity and valence, the *target* of the news also matters. That is, how minority individuals are represented and typified in the media can uniquely shape the public's evaluations of specific group memberships. Group membership might be defined in terms of the country (e.g., Albanian vs. Moroccan minorities) or continent (e.g., European vs. African minorities) of origin or the status (e.g., refugee vs. undocumented migrant) assigned to the individual.

Prior research has highlighted that media salience of specific historical events (e.g., terrorist attacks and migration waves) can shape group‐specific attitudes rather than the general perceptions of ethnic diversity (Bobba, Thijs, & Crocetti, 2024). For instance, after the media account of the Islamist terrorism hitting several European (e.g., the 2015/2016 New Year's attacks in Germany; Czymara & Schmidt‐Catran, 2017) and non‐European countries (e.g., Bali in 2002; Legewie, 2013), negative attitudes toward migrants from the Middle East and Africa significantly increased among the German population. Similarly, the media portrayals of Black British as the main actors in the riots that occurred in England in 2011 led to higher threat perceptions and more negative attitudes toward this ethnic minority group (de Rooij et al., 2015).

While prior research has focused on ethnic minority groups typified in terms of their native country or religious background, less is known about the effect of media framing minorities based on their status (i.e., migrants, refugees, and foreigners) in the host country. For instance, while the term *migrants* generally denotes people who change their country of residence, the term *refugees* refers specifically to those who have to leave their country to escape persecution, conflict, or other circumstances and who require international protection (OHCHR, 2020; UNHCR, 2023a). Conversely, the term *foreigners* more broadly refers to individuals born in a country different from the one they live or are currently staying in. This general term is used to denote anyone who is not member of the ethnic majority group, thus implying clear‐cut boundaries between ingroup and outgroup (Essed & Trienekens, 2008; Findor et al., 2021). These terms have various connotations as they remind to specific statuses and rights. Refugees and asylum seekers usually enjoy greater privileges and higher status than unauthorized migrants (Murray & Marx, 2013). For instance, by law, officially registered asylum seekers in Italy have access to several services (e.g., accommodation in reception centers, access to legal and medical support; UNHCR, 2023b) that are usually not provided for the general migrant population. Moreover, prior research has highlighted that attitudes toward refugees remained positive even when openness toward migrants in general declined (Czymara & Schmidt‐Catran, 2017). Despite the intergroup implications of using non‐overlapping labels to identify ethnic minorities (e.g., Ommundsen et al., 2014; for a discussion, see Sajjad, 2018), to the best of our knowledge, no previous study has examined how news related to different target groups (i.e., migrants, refugees, and foreigners) are linked to changes in prejudice.

## Ethnic prejudice and the news environment in Italy

The current study was conducted in the Italian context, which is becoming more ethnically and culturally diverse due to migration flows. Specifically, from the 1980s, Italy has changed from a country of emigration into one of the major destination land for migrants in Europe (United Nations, 2019). As a result, in the Italian context, the term ethnic minorities refers to individuals who have a migrant background because they are either born abroad (e.g., first‐generation migrants or refugees) or have at least one parent born abroad (i.e., second‐generation migrants). In contrast, and differently from countries with a longer migration history, third‐generation migrants are quite rare, especially among adolescents (as evident from other research conducted on ethnic minority youth population in Italy; e.g., Borraccino et al., 2018; Palladino et al., 2020).

Currently, 5 million ethnic minority individuals (i.e., 8.5% of the entire population) live in Italy (ISTAT, 2023), one‐fifth of which are minors. Relatedly, 10.4% of the general student population in Italy is made up of ethnic minority students who were either born in Italy from ethnic minority parents (i.e., second‐generation; 6.8%) or abroad (i.e., first‐generation; 3.6%) (Ministero della Pubblica Istruzione, 2021). Interestingly, the percentage of ethnic minority students across all educational levels (i.e., primary to secondary school) rises to 17.0% in the Emilia‐Romagna region, where the current study was conducted. A similar pattern can be observed when focusing exclusively on secondary high schools, with 13.5% of ethnic minorities within the Emilia‐Romagna high school's student population compared to the country's average (i.e., 8%; Ministero della Pubblica Istruzione, 2022). Thus, this region was chosen as the most ethnic and culturally diverse in the Italian context.

This increasing diversification of Italian society might offer novel and more frequent opportunities for contact and interactions between ethnic majority and minority individuals (Allport, 1954). However, the positive implications of ethnic diversity can be hindered by social and political conditions that impede the cohesion of society. Relatedly, Italy has generally adopted a “temporary integration” approach to deal with recent increases in migration flows, which sets different standards and conditions for ethnic minority and majority individuals. Specifically, on the one hand, migrants (and their children) have access to fundamental rights (e.g., education and health) in a way that equates them to native Italians. However, they also face obstacles in their political participation and access to nationality, conditions that relegate them to the status of foreigners (MIPEX, 2020). For instance, from both a cultural and legislative point of view, Italian citizenship is almost exclusively based on blood ties (i.e., *ius sanguinis* principle) and can be acquired either by birth (i.e., having one parent with Italian citizenship) or marriage. Children with a migrant descent who were born in Italy can ask for Italian citizenship when they come of age, but the success of this process is not guaranteed due to stringent criteria and bureaucratic complexities. These conditions limit the chances of youth and adults with a migrant background to be recognized as Italian citizens (Colombo & Rebughini, 2013).

The Italian news environment offers representations of ethnic minorities that usually align with these political and social dichotomies (De Rosa et al., 2023) and might orient how ethnic majority Italian adolescents think and feel about ethnic minorities (Maratia et al., 2023). Specifically, the news provided in national newspapers is replicated across multiple media sources, such as in the news press on television and the social media channels of the newspapers, thus fully reflecting the general information environment that characterizes this national context. Interestingly, prior research with Italian adolescents has highlighted that the salience of current events (i.e., Ukranian war) in traditional media sources (i.e., newspaper) is significantly linked to changes in youth's prejudice (Bobba, Thijs, & Crocetti, 2024). The same effect, however, was not found for media salience on Twitter, possibly because this social media is not the preferred platform among Italian adolescents (Marengo et al., 2022).

## The present study

The purpose of this study was to unravel how different features of the information environment are associated with changes in adolescents' ethnic prejudice. First, it aims to examine the associations between the quantity of news about ethnic minorities and changes in prejudice in adolescence. In line with prior research (e.g., Visintin et al., 2017), the amount of news about ethnic minority individuals is expected to be related to increased adolescents' negative emotions and feelings about ethnic minorities (H1). Moreover, as a second goal, it further studies the interplay between valence (i.e., neutral, positive, and negative) of the news and changes in ethnic prejudice. In line with previous findings (e.g., Fuochi et al., 2020; Schemer, 2012), positive and negative representations of ethnic minorities in the newspaper are expected to respectively be associated with decreased (H2a) and increased (H2b) prejudice levels of adolescents, while no expectations were set out for what concerns neutral news. Third, this study aims to investigate whether the target of the news (i.e., migrants, refugees, and foreigners) has a role in changes in ethnic prejudice. Considering the higher status usually assigned to refugees compared to other groups of minorities (e.g., Czymara & Schmidt‐Catran, 2017; Murray & Marx, 2013), negative news concerning this target outgroup is expected not to be associated with prejudice levels, whereas negative news about migrants and foreigners is expected to be linked to heightened prejudice (H3a). Similarly, positive news about refugees is expected to be associated with greater reductions in prejudice compared to positive news about migrants and foreigners in general (H3b). All these hypotheses will be tested examining both the affective and cognitive dimensions of prejudice. The fourth and last goal of the current study was to explore the moderating role of adolescents' direct news consumption on the associations between news features (i.e., quantity, valence, and target) and ethnic prejudice.

## METHODS

### Participants

Data for this study are drawn from the ongoing longitudinal project IDENTITIES “Managing identities in diverse societies: A developmental intergroup perspective with adolescents,” a cohort sequential research conducted in the Northern part of Italy (i.e., the Emilia‐Romagna region). Specifically, 962 adolescents (at baseline: *M*_age_ = 15.67, SD_age_ = 1.17, 48.13% females) attending the first and third year of 14 high schools located in the Northern part of Italy (i.e., Emilia‐Romagna region) were included in the current study. Since the focus was on prejudice against ethnic minorities, only ethnic majority Italian adolescents (i.e., whose parents were both born in Italy) were included in the current study. Thus, adolescents of migrant descent were not included in this study. Additional details on the sample characteristics are provided in Table 1.

**TABLE 1:** Demographic characteristics of the sample.

Sample characteristics (at T1)	
Total sample (*N*)	962
Sex (%)	
Female	48.13
Male	51.87
Age (*M* (SD))	15.67 (1.17) years
Age cohort (%)	
First year of high school	49.11
Third year of high school	50.89
School track (%)	
Academic‐oriented	47.14
Technical school	32.88
Vocational school	19.98
Mother's educational level (%)	
High (i.e., University degree or higher)	34.49
Medium (i.e., high school diploma)	49.33
Low (i.e., up to middle school diploma)	16.18
Father's educational level (%)	
High (i.e., University degree or higher)	25.25
Medium (i.e., high school diploma)	47.70
Low (i.e., up to middle school diploma)	27.05

Abbreviations: *M*, mean; SD, standard deviation; T, Time.

For the current study, participants completed questionnaires at the end of one school year (i.e., April/May 2022, T1) and at the beginning of the following school year (i.e., September/October 2022, T2). Most adolescents participated in the first (88.56%) and second (80.56%) assessments. Attrition checks revealed that youth who participated in only one of the two assessments displayed slightly higher levels of affective (*F* = 21.35, *p* < .001; η^2^ = .02) and cognitive (*F* = 14.99, *p* < .001; η^2^ = .02) prejudice at T1, although the dimension of these effects were quite small. Conversely, no significant differences between the two groups of participants emerged on their levels of affective (*F* = 1.66, *p* = .198; η^2^ = .00) and cognitive (*F* = 0.31, *p* = .575; η^2^ = .00) prejudice at T2. The questionnaire completion rate was high within each assessment (95.54% at T1 and 94.58% at T2). Therefore, missingness was mostly due to participants not filling out the questionnaire, mainly because they were not in school on the day of data collection. Little's (1988) Missing Completely at Random (MCAR) test yielded a normed χ^2^ (χ^2^/df = 884.57/499) of 1.77, indicating that data were likely missing completely at random. Therefore, the total sample of 962 participants was included in the analyses, and missing data were handled with the Full Information Maximum Likelihood (FIML) option available in M*plus* (Kelloway, 2015).

### Procedure

The present study was approved by the Ethics Committee of the Alma Mater Studiorum University of Bologna (Italy) as part of the ERC‐Consolidator project IDENTITIES “Managing identities in diverse societies: A developmental intergroup perspective with adolescents.” This research involves a target group of adolescents from several high schools. Schools were selected through a stratified (by track and level of urbanization) randomized method, and principals were approached to present the project. Upon their approval, the study was presented to students and their parents, who also received detailed oral and written information. Active consent from parents was obtained before their children's participation. Active consent was also obtained from adolescents of age while their underage peers provided their assent to participate in the project. Participation in the study was voluntary, and students were informed that they could withdraw their consent at any time. The IDENTITIES project started in 2022 and includes multiple annual, monthly, and daily assessments. At each wave, adolescents completed an online questionnaire during class hours. They were required to create a personal code to ensure confidentiality and pair their answers over time.

### Measures

#### Demographics

Participants' socio‐demographic information (e.g., age, gender, and parents' educational level) was collected at the beginning of the study.

#### Affective prejudice

Feelings toward the most represented ethnic minority groups in the Italian context (i.e., Albanian, Romanian, Moroccan, Chinese, and Ukrainian; ISTAT, 2020) were measured at each time point using the feeling thermometer scale (Haddock et al., 1993; for use of the Italian version, see Bobba & Crocetti, 2022). This measure asks participants to rate how much they like members of different ethnic groups on a scale from 0° (not at all) to 100° (very much). To simplify the presentation of results, items were reversed so that the higher the score, the higher the level of affective prejudice. A total affective prejudice score was computed using the mean level of liking expressed for these different outgroups. Cronbach's Alphas were .91 and .93 at T1 and T2, respectively.

#### Cognitive prejudice

To evaluate the cognitive component of prejudice, five items were adapted from Brown et al. (2008). Adolescents rated their agreement on a 5‐point Likert scale (from 1 “completely disagree” to 5 “completely agree”). A sample item is the following: “Foreign people should be marginalized in Italian society.” Cronbach's Alphas were .88 and .89 at T1 and T2, respectively.

#### Newspaper consumption

The extent to which adolescents relied on newspapers as a source of information was assessed at T1 with two items (“How often do you use local newspaper to keep yourself updated with daily news?” and “How often do you use national newspaper to keep yourself updated with daily news?”). Ratings were expressed on a 7‐point Likert‐type scale from 1 (never) to 7 (more than once a day). On average, adolescents indicated that they rarely rely on newspapers as a source of information (*M* = 1.65, SD = 1.04). To test moderation using a multigroup approach, participants were assigned to either the low (68.93%; *M*_low_ = 1.09, SD = 0.19) or the high (31.07%; *M*_high_ = 2.90, SD = 1.08) newspaper consumption groups based on their score below or above the sample mean (i.e., no participant had a score equivalent to the sample mean score).

#### Newspaper data

The current study focused on the Italian national newspaper La Repubblica to examine the quantity, valence, and target of the news about ethnic minorities. This media outlet represents the second most widespread daily newspaper in the country and the first in the area of the study (i.e., the Emilia‐Romagna region; ASD, 2022). As a preliminary step, the digital daily newspapers of the 7 days prior to the T2 assessment (i.e., end of September to beginning of November 2022) were downloaded and screened using three keywords: migr* (to identify articles about “migrante/i”, Italian for “migrant(s)”, and “migrazione”, Italian for “migration”), stranier* (i.e., Italian for “foreign*”), and rifugiat* (i.e., Italian for “refugee*”). News articles including any of the selected keywords were read, and the authors extracted three different types of scores (i.e., quantity alone, valence alone, valence and target combined). First, regarding quantity, for each participant, a *quantity of news score* was computed as the average amount of news about migrants, refugees, and foreigners over the week preceding the completion of the T2 questionnaire. Second, regarding valence alone, for each newspaper the content of the sentence(s) containing the target terms migrant*, foreign*, and refugee* was evaluated as either neutral (i.e., reporting general statements or objective information), positive (i.e., reporting positive or sympathetic views about the outgroup), or negative (i.e., reporting statements against migration or stereotypical representations of ethnic minorities). For each participant, three *news valence scores* were computed as the weekly average amount of neutral, positive, and negative news, separately. Last, regarding valence and target combined, the valence assessment was applied separately for each target (i.e., migrants, foreigners, refugees). This allowed to compute nine *news target scores* for each participant obtained as the weekly average amount of neutral, positive, and negative news about migrants, refugees, and foreigners separately. It should be noted that, due to the numerosity and regional distribution of the sample within the region of the study, the T2 data collection lasted 5 weeks, and adolescents from different schools completed their questionnaires at different moments across this period. This means that youth who completed the T2 assessment on the same day also shared the same newspaper data. Further details on the variability in newspaper data, newspaper data extraction procedure, and examples of the coded articles (see Table S1) are provided in document S1 of the Supplemental Materials. Initially, 20% of the articles were screened and coded independently by the first and second authors. Disagreements were solved upon discussion among the coders. Intraclass correlation coefficients were high across all data extracted, ranging from .73 (for negative news about foreigners) to .98 (for neutral news about migrants). The agreement was also estimated by means of the Krippendorff's alpha (De Swert, 2012) which consistently indicated an agreement rate ranging from .57 (for negative news about foreigners) to .90 (for neutral news about migrants). Therefore, the remaining news articles were screened and coded by the second author alone.

### Strategy of analysis

Descriptive statistics and correlations were computed using IBM SPSS Version 29.0 for Windows. The remaining analyses were performed in M*plus* 8.10 (Muthén & Muthén, 2017) using the Robust Maximum Likelihood (MLR) estimator. As a preliminary step, longitudinal measurement invariance was tested separately for the affective and cognitive ethnic prejudice scales. This procedure is further detailed in document S4 of the Supplemental Materials.

To address the main goals of the current study, several linear regression models with observed variables were tested in M*plus*, and model fit was assessed based on multiple criteria. Specifically, the Comparative Fit Index (CFI) with values higher than .90 and .95, combined with the Root Mean Square Error of Approximation (RMSEA) and the Standardized Root Mean Residual (SRMR), with values below .08 and .05 indicate an acceptable and excellent fit of the model (Byrne, 2012). Additionally, the RMSEA's 90% confidence interval's upper bound lower than .10 suggests an acceptable fit of the model (Chen et al., 2008). In each model, newspaper data were entered as predictors of affective and cognitive prejudice at T2, controlling for prejudice levels at T1. Additionally, each model included participants' sex (Male = 0, Female = 1), their school track (Academic‐oriented = 0, Technical and vocational combined = 1), and their parents' educational level (which was obtained as the sum of mother's and father's educational level) as covariates.

Regarding the first goal of the study, Model 1 examined the associations between news quantity and adolescents' prejudice. To address the second goal, in Model 2, the number of neutral, positive, and negative news were simultaneously entered as predictors of affective and cognitive prejudice. Concerning the third goal, three regression models were performed separately for neutral (Model 3a), positive (Model 3b), and negative (Model 3c) news, in which newspaper data about migrants, refugees, and foreigners were included as predictors of youth's prejudice. Last, to address the fourth and last aim, these main models were replicated in a multigroup framework to examine the moderating role of newspaper consumption. For each multigroup model, the Wald test was applied to identify significant differences in regression paths between youth in the low and those in the high newspaper consumption groups. This strategy aligns with the approach undertaken in related research on media salience and ethnic prejudice among adolescents (Bobba, Thijs, & Crocetti, 2024).

## RESULTS

### Preliminary analyses

Means, standard deviations, and correlations among the main study variables are reported in Tables S2 and S3 of the Supplemental Materials. Results of the longitudinal measurement invariance are reported in Table S4 of the Supplemental Materials. Full metric invariance was reached for both affective and cognitive prejudice scales, and therefore, we could proceed with the main analyses. Analyses codes and outputs can be retrieved from: https://osf.io/6btah/.

### The role of news quantity, valence, and target on adolescents' prejudice

The current study sought to examine the interplay between the quantity (first goal; Model 1), valence (second goal; Model 2), and target (third goal; Models 3a–c) of the news about ethnic minority groups and changes in ethnic prejudice in adolescence. Fit indices of the regression models are reported in Table 2. All models displayed a reasonable fit, although the RMSEA and SRMR were close to the cutoff values. Models explained a great portion of variance both in affective (around 56%) and cognitive (around 47%) ethnic prejudice.

**TABLE 2:** Model fit indices of the regression models.

Models	Model fit				
	χ_SB_^2^	df	CFI	SRMR	RMSEA [90% CI]
M1: News quantity model	101.134	10	.892	.093	.101 [.084, .120]
M2: News valence model	114.211	14	.887	.086	.090 [.075, .105]
M3: News target model					
M3a: Neutral news model	114.600	14	.888	.090	.090 [.075, .106]
M3b: Positive news model	115.677	14	.885	.087	.090 [.076, .106]
M3b: Negative news model	111.095	14	.891	.091	.088 [.074, .104]

Abbreviations: CFI, Comparative Fit Index; CI, confidence interval; df, degree of freedom; RMSEA, Root Mean Square Error of Approximation; SRMR, Standardized Root Mean Square Residual; χ_SB_^2^, Satorra–Bentler scaled chi‐square.

Results regarding the role of news *quantity* are reported in Table 3. Contrary to our hypothesis, the amount of news about ethnic minorities was not significantly linked to changes in affective and cognitive prejudice. Regarding news *valence* (Table 3), neutral news was not related to changes in prejudice levels over time. Conversely, positive news was associated with reduced, while negative news was linked to increased cognitive ethnic prejudice, while no effects emerged for its affective counterpart. Last, regarding news *target* (Table 4), we found a few significant effects of news only on the cognitive dimension of prejudice. Specifically, neutral news concerning the outgroup of foreigners, but not those about migrants and refugees, were linked to heightened levels of cognitive prejudice. Interestingly, positive news displayed different effects on ethnic prejudice levels depending on the target. Positive news about migrants was linked to lower, while positive news about refugees was associated with higher cognitive prejudice levels. No effect emerged for negatively‐valenced news, regardless of their target.

**TABLE 3:** News quantity and valence models.

	Affective prejudice T2 *β* (*SE*)	Cognitive prejudice T2 *β* (*SE*)
M1: News quantity model		
Prejudice T1^a^	.733 (.024)***	.668 (.034)***
Sex	−.064 (.027)*	−.137 (.029)***
Parents' educational level	−.062 (.026)*	−.036 (.029)
School track	.103 (.029)***	.015 (.031)
News Quantity	−.024 (.026)	−.013 (.029)
*R* ^2^	.562 (.032)***	.467 (.042)***
M2: News valence model		
Prejudice T1^a^	.732 (.024)***	.664 (.035)***
Sex	−.062 (.027)*	−.128 (.029)***
Parents' educational level	−.060 (.027)*	−.029 (.030)
School track	.101 (.032)**	−.031 (.035)
Neutral News	.016 (.106)	−.004 (.112)
Positive News	−.046 (.101)	−.341 (.108)**
Negative News	.006 (.114)	.330 (.129)*
*R* ^2^	.561 (.032)***	.470 (.042)***

*Note*: Sex: 0 = Male, 1 = Female. Parents' educational level was calculated as the sum of mother and father educational level, so that the higher the value, the higher the educational level of both parents combined. School track: 0 = Academic‐oriented track, 1 = Technical and vocational tracks combined. Abbreviations: *SE*, Standard error; T, Time; *β*, Standardized regression coefficient.

^a^ Affective prejudice at T1 and cognitive prejudice at T1 were entered as predictors of affective prejudice at T2 and cognitive prejudice at T2, respectively.

*p < .05; **p < .01; ***p < .001.

**TABLE 4:** Standardized regression coefficients: News targets by valence models.

	Affective prejudice T2 *β* (*SE*)	Cognitive prejudice T2 *β* (*SE*)
M3a: Neutral news model		
Prejudice T1^a^	.733 (.024)***	.666 (.034)***
Sex	−.062 (.027)*	−.135 (.029)***
Parents' educational level	−.058 (.027)*	−.026 (.030)
School track	.098 (.032)**	−.011 (.033)
News about migrants	−.023 (.046)	−.056 (.051)
News about refugees	.015 (.039)	.011 (.041)
News about foreigners	.016 (.035)	.081 (.036)*
*R* ^2^	.562 (.032)***	.468 (.043)***
M3b: Positive news model		
Prejudice T1^a^	.733 (.024)***	.664 (.035)***
Sex	−.063 (.027)*	−.128 (.029)***
Parents' educational level	−.059 (.027)*	−.021 (.030)
School track	.096 (.033)**	−.025 (.034)
News about migrants	−.044 (.064)	−.205 (.069)**
News about refugees	.010 (.044)	.140 (.045)**
News about foreigners	−.012 (.046)	−.080 (.049)
*R* ^2^	.561 (.032)***	.469 (.042)***
M3c: Negative news model		
Prejudice T1^a^	.733 (.024)***	.665 (.035)***
Sex	−.063 (.027)*	−.128 (.029)***
Parents' educational level	−.060 (.027)*	−.033 (.030)
School track	.103 (.031)**	−.021 (.034)
News about migrants	−.036 (.052)	−.037 (.056)
News about refugees	−.001 (.035)	.073 (.039)
News about foreigners	−.017 (.056)	−.047 (.058)
*R* ^2^	.561 (.032)***	.470 (.042)***

*Note*: Sex: 0 = Male, 1 = Female. Parents' educational level was calculated as the sum of mother and father educational level, so that the higher the value, the higher the educational level of both parents combined. School track: 0 = Academic‐oriented track, 1 = Technical and vocational tracks combined. Abbreviations: *SE*, Standard error; T, Time; *β*, Standardized regression coefficient.

^a^ Affective prejudice at T1 and cognitive prejudice T1 were entered as predictors of affective prejudice at T2 and cognitive prejudice at T2, respectively.

*p < .05; **p < .01; ***p < .001.

Covariates displayed a significant and similar pattern of association with prejudice across all models. Specifically, girls reported significant decreases in both affective and cognitive prejudice over time. Moreover, students attending technical and vocational high schools displayed higher affective prejudice compared to those attending academic‐oriented tracks. Last, parents' educational level was negatively linked to affective prejudice.

### The moderating role of direct newspaper consumption

The last goal of the current study was to test whether youth's direct consumption of the media outlet would moderate the associations between features of the news and prejudice. Multigroup analyses revealed that adolescents with low and those with high newspaper consumption did not significantly differ in the significance nor strength of associations between news quantity, valence, and target and changes in affective and cognitive ethnic prejudice (see Table S5 of the Supplemental Materials). Therefore, the extent to which youth relied on newspapers to keep themselves informed did not significantly moderate the news‐prejudice links.

## DISCUSSION

In line with the information environment framework (Boomgaarden & Vliegenthart, 2009), the current research examined how multiple features (i.e., quantity, valence, and target) of the news about ethnic minorities can create a shared corpus of macro‐contextual representations that inform adolescents' affective and cognitive prejudice. Overall, these findings provide novel insight into the way media content and framing of ethnic minorities are linked to changes in prejudice at this developmental stage. Such knowledge is crucial not only to advance our theoretical understanding of prejudice determinants but also to build evidence‐based interventions that are sensitive to the cultural, mediatic, and historical influences at play in a given context (Beelmann & Lutterbach, 2021).

### Quality over quantity: The associations between news quantity and valence and ethnic prejudice

The first and second goals of the current study were to examine whether the quantity and valence of news about ethnic minorities would be associated with changes in affective and cognitive prejudice among adolescents. Regarding news quantity, this study found that higher exposure to news about ethnic minorities in the previous week does not translate into a change in adolescents' levels of ethnic prejudice. The quantity of news about ethnic minorities can be considered an expression of the salience of this topic within the general media environment. The salience of this topic comprises a mix of neutral, positive, and negative information that can ultimately give rise to opposite framings (e.g., as a threat or as a victim) of this target group. Consequently, the quantity of news alone might not be able to skew adolescents' perceptions about ethnic minorities. This lack of findings on the role of quantity also aligns with research on intergroup contact, which has stressed the importance of quality over quantity in orienting attitudes (e.g., De Coninck et al., 2021) and behaviors (e.g., Johnston & Glasford, 2018). Relatedly, the current research found that the effects of media depended on the valence of the news reported. Specifically, positive and negative news were associated with reduced and increased cognitive ethnic prejudice, respectively.

In line with the valence framing theory (Boomgaarden, 2007) and prior experimental findings (e.g., Fuochi et al., 2020; Schemer, 2012), the current study highlights how differently‐valenced narratives about ethnic minorities can be intertwined with individuals' thoughts. However, the current study highlights that valenced news is strongly associated with prejudice regardless of individuals' direct exposure to the media content. That is, an information environment characterized by positive framing and supportive discourses about migration might elicit empathetic feelings toward the outgroup, which in turn have been previously linked to lower levels of prejudice (Bobba & Crocetti, 2022; Miklikowska, 2018). Similarly, positively framing ethnic minorities might contribute to reductions in threat perceptions (e.g., Lissitsa et al., 2023) and increased trust (e.g., Visintin et al., 2017), which consequently lead to more positive beliefs about members of the outgroup.

In contrast, negative news about minorities increases threat perceptions (Esses et al., 2013; Lissitsa et al., 2023; Schlueter & Davidov, 2013) and intergroup anxiety (Visintin et al., 2017) and offers stereotypical negative representations of migrants. These, in turn, prime youth's perceptions of and later judgments about diverse others, thus exerting an effect on their cognitive prejudice (Intravia & Pickett, 2019). Moreover, the current findings align with prior experimental research (Lissitsa et al., 2023) in highlighting that neutrally‐valenced news reports are not linked to changes in adolescents' perceptions of ethnic minorities. That is, the information environment created by news reports is intertwined with youth's prejudice only when positively or negatively valenced, as these depictions can contribute to forming and consolidating specific thoughts about and representations of ethnic minorities (Lambert et al., 2010). Overall, these findings build upon and extend prior literature on the effect of direct media exposure on ethnic prejudice by highlighting that the news quantity and valence define an overarching information environment that is linked with the development of intergroup attitudes and relations in adolescence. However, neutrally, positively, and negatively valenced media reports might have more nuanced associations with changes in ethnic prejudice when examined in combination with the subject of such news. This suggests the importance of taking into account the target of the news to get a comprehensive understanding of this distal correlate of prejudice.

### The target matters: The interplay between valence and target status on prejudice

The current study extends prior literature by examining whether neutral, positive, and negative news would be differently linked to affective and cognitive prejudice levels also depending on the status (i.e., migrant, refugee, and foreigner) assigned to the news target. The findings highlighted a nuanced pattern of associations between news representations and changes in prejudice in adolescence, but only for neutral and positive news. Conversely, negative news about migrants, refugees, and foreigners alike were not significantly associated with changes in affective or cognitive prejudice.

Interestingly, neutral news were linked to heightened levels of cognitive prejudice only when they concerned the group of foreigners. This finding might be related to the fact that the term foreigner represents a broad and de‐personalizing category (Asbrock et al., 2014), which in turn makes intergroup differences salient even when factual reports are presented in the news. That is, the use of such a de‐individuating category might lead the information environment to create and enhance a dichotomous perception of society as “Us vs. Them,” which is at the core of ethnic prejudice and discrimination (Brown, 2011). On the contrary, relying on individuating representations of ethnic minorities supports the process of decategorization and the reduction of intergroup biases (Albarello et al., 2023; Prati et al., 2021). Prior research has documented how prejudice can be either reinforced or reduced through the use of language. Specifically, nouns more strongly contribute to impression formation because they identify the general category to which an individual or element belong and automatically extend category‐congruent representations from the group to the single (Carnaghi et al., 2008). Relatedly, experimental studies have found that using nouns rather than adjectives to define ethnic categories leads to more pronounced intergroup bias (Graf et al., 2013, 2020).

Positively valenced news were linked to different effects on prejudice based on the target. On the one hand, positive news about migrants was found to be significantly linked with reduced cognitive prejudice. On the other hand, positive news about refugees had the opposite effect, as they were associated with increased cognitive ethnic prejudice. This opposite implications of news based on the target might appear quite surprising in light of prior research. For instance, previous studies have usually highlighted a more positive conception of refugees compared to other groups of migrants (e.g., Czymara & Schmidt‐Catran, 2017; De Coninck et al., 2021). These findings have been interpreted as a result of hierarchies of deservingness between different groups of migrants, with refugees being perceived as (more) entitled to protection, rights, and resources from both a legislative perspective and within the public discourses (Sajjad, 2018).

However, recent comparative research has highlighted a strong shift in attitudes toward the group of refugees at the beginning and during the 2015 “refugee crisis.” The concept of refugee as a person entitled to support and help was prevalent at the beginning of this historical moment, leading to more positive evaluations toward this minority group. Subsequently, however, people's solidarity decreased as the crisis unfolded, possibly as a consequence of a negative contamination of the refugee concept, which the public discourse has frequently equated to that of economic migrants with no humanitarian reasons for moving country (Janky, 2019; Verkuyten et al., 2018). Such contamination of labels and meanings has been mostly traced back to far‐right political discourses that emphasize migration‐related threats with the aim of controlling national borders and limiting the share of resources (Sajjad, 2018). In line with this, recent research on the implications of labeling has found that attitudes toward “refugees” were significantly less positive compared to those toward individuals labeled as “migrants” (Graf et al., 2023) and “foreigners” (Findor et al., 2021) across several European countries.

Besides the role of political debates and shared representations, the decline in sympathetic and humanitarian feelings toward the group of refugees has also been read in light of the threat‐benefit model. This theoretical framework underlines that outgroup members are not only perceived in terms of threat, but also in relation to the benefits they might bring to the ingroup and larger society (for further details, see Tartakovsky & Walsh, 2016). Along this line, Graf et al. (2023) found benefit perceptions to mediate the labeling‐attitude link, with participants displaying more positive attitudes toward migrants compared to refugees because they evaluated the former as more beneficial for the receiving country. Therefore, far from being surprising or counterintuitive, the negative association between positive news about refugees and cognitive prejudice found in the current study can be interpreted against this background and in line with empirical research conducted during and after the “refugee crisis.” This historical circumstance appears to have changed the meanings associated with specific labels and the wider implications of language and news content. Positive representations of refugees in the news might reinforce the perception of these individuals as lacking any form of personal agency and active participation in society (Chouliaraki & Zaborowski, 2017) and therefore not able to bring any tangible benefit to the destination country.

Another possible explanation for the present findings might be that the news depictions of refugees, although positively valenced and sympathetic in their tone, might increase ethnic prejudice by heightening the salience of stereotypical and de‐personalized views of the outgroup (Kyriakidou, 2021). This kind of representation highlights a paternalistic or benevolent form of prejudice, whereby groups characterized by high warmth and low competence are often pitied but also actively ignored and not respected (Cuddy et al., 2007; Fiske et al., 2002). All in all, current and previous findings warrant the need for more studies on the labeling processes occurring in the media and public discourses. For instance, future research could analyze the content of language more in‐depth to understand whether benevolent representations of ethnic minorities or of specific target outgroups might differently contribute to prejudice development. Furthermore, greater attention should be paid to the role of context and the normative, institutional, and cultural influences at play (Findor et al., 2021).

### From the macro‐context to the individual: The role of the information environment

Last, the current study aimed to advance the theoretical understanding of media influences by examining whether the effect of quantity, valence, and target of the news about ethnic minorities on changes in prejudice would hold regardless of adolescents' actual consumption of and exposure to the newspaper. In line with expectations and confirming the assumption of the information environment theory (Boomgaarden & Vliegenthart, 2009; Weimann & Brosius, 1994) and findings from prior research (Bobba, Thijs, & Crocetti, 2024), the associations between news data and changes in prejudice were equal across adolescents who rarely and those who usually read newspaper. This is possibly a consequence of the fact that information provided in national newspapers is replicated across multiple channels (e.g., news press, television) and transmitted via interpersonal communication. These conditions contribute to creating a shared corpus of representations and knowledge that can influence not only those who are directly exposed to the news, but also those who receive this information indirectly.

These findings highlight the important role played by the information environment in contributing to the understanding of prejudice. Despite being positioned in the most distal layer of the ecological developmental context, media can set the conditions under which ethnic prejudice changes and youth form and consolidate their feelings and beliefs about others (Lecheler et al., 2019). Moreover, the information environment often appears to be nuanced both in terms of valence and framing of ethnic minorities, and these subtleties of language can shift youth's prejudice levels in opposite, and sometimes unexpected, directions.

### Thinking over feeling: The unique implications of news for cognitive prejudice

In line with a multidimensional definition of ethnic prejudice (Brown, 2011), the current study examined whether the quantity, valence, and target of the news about ethnic minorities would be associated with changes in both affective and cognitive prejudice. Interestingly, news features were exclusively associated with changes in the cognitive dimension, while no significant effect emerged for its affective counterpart. This finding is in contrast with previous research highlighting that exposure to valenced news about migration was significantly linked to the affective dimension of prejudice (Graf et al., 2020). Additionally, it deviates from findings on the implications of media salience of the Russia‐Ukraine war for changes in adolescents' affective prejudice (Bobba, Thijs, & Crocetti, 2024). However, it should be noted that none of these studies included a measure of cognitive prejudice, thus limiting the comparison with the current findings.

The affective dimension of prejudice tackles individuals' (negative) feelings toward ethnic minorities, which might be more susceptible to the influence of direct intergroup encounters and experiences in the proximal contexts of development. For instance, prior meta‐analytic findings highlighted that the prejudice‐contact link was stronger for indicators of affective compared to cognitive prejudice (Tropp & Pettigrew, 2005). Conversely, distal influences, such as those pertaining to the information environment, might contribute to more substantial changes in cognitive representations of the outgroup and related stereotypes. Along this line, media representations of ethnic minorities might be a source of information and priming about members of this group (Dixon & Azocar, 2007; Mastro & Tukachinsky, 2011). In turn, this priming process might guide the ways in which youth think about ethnic minorities and lead to the attribution of negative stereotypes and representations to them.

### Theoretical and practical implications

Overall, findings from the current study have important theoretical and practical implications. From a theoretical standpoint, they lend support to the premises of the information environment approach (Boomgaarden & Vliegenthart, 2009) and extend its implications to the study of adolescents' ethnic prejudice development. Specifically, the current findings suggest that the labels used to define ethnic minorities can reinforce dichotomous views of ingroup and outgroups, setting borders (Chouliaraki & Zaborowski, 2017), and hierarchies of deservingness (Kyriakidou, 2021) rather than providing humanizing representations able to create a sense of common identity. A possible solution to this issue might be the inclusion of personal stories that humanize individuals rather than focusing on anonymized groups, which can help the audience empathize with the experiences of others (Birks, 2017; Pantti & Ojala, 2019). Furthermore, this study highlights the differential interplay between affective and cognitive prejudice and news features. This means that future research should strive to account for the multifaceted nature of prejudice, given affective and cognitive prejudices' unique developmental patterns (for a review, see Crocetti et al., 2021) and associations with proximal and distal factors (e.g., Bobba, Branje, & Crocetti, 2024; Thijs et al., 2016; for a review, see Beelmann & Heinemann, 2014).

From a practical point of view, this study suggests the need for interventions that account for the complex contextual, mediatic, and historical influences at play in a given moment (Beelmann & Lutterbach, 2021) and that exploit the full potential of these macro‐contextual factors. For instance, media literacy education curricula (e.g., Paluck, 2009; Ramasubramanian, 2007; for a review, see Scharrer & Ramasubramanian, 2015) might be effective in challenging stereotypical and negative representations of ethnic minority groups in the media. In turn, these interventions can prevent the negative consequences of being exposed to an information environment that heightens differences rather than similarities, ultimately supporting youth's positive adjustment and the stability and cohesion of current multicultural societies (Koopmans & Schaeffer, 2016).

### Limitations and future research suggestions

Findings from the current study should be read in the light of some limitations. First of all, this research relied on a single media source, mainly a national newspaper, to identify the quantity, valence, and target of news about ethnic minorities. However, the information environment of current society is shaped by both traditional (i.e., newspaper and television) and modern (i.e., social media and online news) media sources, which together might change the macro‐contextual conditions to which youth are exposed.

Second, this study examined the quantity, valence, and target of the news over a relatively short period (i.e., 1 week) and how it might shape changes in levels of ethnic prejudice. Future research could benefit from examining multiple media sources over a longer time span. This would allow unraveling the short‐ and long‐term effects of the information environment surrounding adolescents and understanding whether content replicated across multiple media sources can differently impact intergroup relationships.

Moreover, this study did not examine the processes through which the media narratives shape the development of affective and cognitive prejudice. Specifically, the information environment might also influence the characteristics of the proximal contexts (e.g., parents' ethnic prejudice, school, and neighborhood) within which youth are embedded. For instance, it might be that the media content is linked to adults' prejudice levels, which in turn are transmitted to their children via processes of unidirectional or reciprocal influences. Future studies are needed to unravel the unique and combined (i.e., meso‐system) effects of the multiple ecological factors at play.

Last, this research involved ethnic majority Italian adolescents living in the region (i.e., Emilia‐Romagna) with the highest percentage of ethnic minority youth within the general and secondary‐school student population. On the one hand, this means that the current study tackled a crucial and fruitful environment where intergroup relationships and attitudes form and consolidate. On the other hand, the unique features of the school and national contexts examined might limit the generalizability of current findings to countries characterized by different socio‐historical, mediatic, and political landscapes.

## CONCLUSION

There is consistent evidence that media and the news can shape adults' attitudes toward ethnic minority groups (e.g., Conzo et al., 2021; Dixon, 2008; Schemer, 2012). This study significantly advances current knowledge by examining their associations with prejudice in the crucial developmental phase of adolescence, in which youth form and change their personal and social views and attitudes. Combining the theories of information environment and ecological development, the current study examined whether quantity, valence (i.e., neutral, positive, negative), and target (i.e., migrant, refugee, foreigner) of the news in the national newspaper would be linked to significant changes in affective and cognitive ethnic prejudice levels. While the total amount of news about ethnic minorities was not significantly associated with prejudice, positively‐ and negatively‐valenced news were linked respectively to decreased and increased levels of cognitive, but not affective, prejudice. Further, neutral and positive news had unique relations with prejudice depending on the news target. These associations were not moderated by youth's levels of direct consumption of the media source examined (i.e., newspaper) supporting the contents of the information environment theoretical approach. Overall, this study suggests that narratives provided in the media have the potential to generate an information environment that is intertwined with public and individual opinions and perceptions.

## AUTHOR CONTRIBUTIONS

B.B. conceived the current study, wrote the manuscript, performed the statistical analyses, and participated in the interpretation of the results, and in drafting and revising the article; A.M. wrote the manuscript, participated in the interpretation of results and in drafting the article; E.C. conceived the current study, wrote the manuscript, participated in the interpretation of the results, and in drafting and revising the article. All authors read and approved the final manuscript and agreed on the authorship order.

## FUNDING INFORMATION

This work was supported by a grant from the European Research Council (ERC) under the Horizon 2020 (H2020) research and innovation framework programme (ERC‐CoG IDENTITIES Grant agreement No. [101002163]; Principal investigator: Elisabetta Crocetti).

## CONFLICT OF INTEREST STATEMENT

The authors report there are no competing interests to declare.

## ETHICS STATEMENT

All procedures performed in this study involving human participants were in accordance with the ethical standards of the Ethics Committee of the Alma Mater Studiorum University of Bologna (Italy) and with the 1964 Helsinki declaration and its later amendments or comparable ethical standards.

## INFORMED CONSENT

Informed active consent was obtained from participants' parents and assent from the participants themselves was also collected.

## Supporting information


Data S1.

## Data Availability

Data, analyses codes, and outputs are publicly available on Open Science Framework and can be retrieved at the following page: https://osf.io/6btah/.
