# Supplementary material for: When ethnic minorities hit the headlines: The longitudinal associations between news features and adolescents' ethnic prejudice
Source: J Res Adolesc. 2024 Sep 3;34(4):1456–70. doi: 10.1111/jora.13013 (PMC11606256; doi:10.1111/jora.13013)
Supplement: Supplementary file 1 — Data S1. [file JORA-34-1456-s001.docx]

**SUPPLEMENTAL MATERIALS**

**S1. Newspaper Data**

**Variability in Newspaper Data and Adolescents’ T2 Assessment**

The current study involved many students from 14 different high schools located in the region of Emilia-Romagna. Given the widespread distribution of participants, the data collection period for each wave was spread across five weeks. Each week, on the same day, the research team visited three schools simultaneously (except for the last week of data collection, when only two schools were visited by the researchers). This means that all the participants in those schools completed their T2 questionnaires on the same day and, consequently, shared the same newspaper data (i.e., data collected over the seven days before the T2 assessment; see below for more information on newspaper data coding and extractions). The distribution of participants across the different weeks ranged from 98 to 216 participants (*M* = 155 participants each week, *SD* = 48.03) at T2.

**Newspaper Data Coding and Extraction**

The current study examined the quantity, valence, and target of the news about ethnic minorities in the Italian national newspaper La Repubblica. Data were extracted manually from the digital versions of the newspaper published daily during the week preceding students’ assessment at T2. The newspapers were screened using the keywords: MIGR*, STRANIER*, and RIFUGIAT*, and subsequently, total and average scores were extracted for each participant and imputed into the main dataset.

The screening process included multiple steps, each conducted separately for every daily newspaper during the data collection period. First, the number of occurrences of the three keywords was summed, and this daily value was stored in an Excel spreadsheet. Second, each sentence containing one of the three keywords was assessed by the authors and coded as either neutral (i.e., reporting general statements or objective information), positive (i.e., reporting positive or sympathetic views about the outgroup), or negative (i.e., reporting statements against immigration or stereotypical representations of ethnic minorities) in valence. The coding process allowed the computation of three daily values (i.e., neutral news, positive news, negative news) obtained as the sum of neutral, positive, and negative sentences about minorities. Third, each sentence was again screened for a combination of valence and target and a total of nine daily scores (i.e., neutral news about migrants, neutral news about foreigners, neutral news about refugees, positive news about migrants, positive news about foreigners, positive news about refugees, negative news about migrants, negative news about foreigners, negative news about refugees) were computed as the sum of occurrences of each combination (e.g., the neutral news migrant score is the sum of all the occurrences of neutral sentences containing the keyword “migrant” in each daily newspaper). Examples of coded news (for valence and target) are available in Table S1.

Total scores for news quantity, valence, and target were subsequently computed for each participant as averages across the seven days before T2 questionnaire completion. Since participants completed the T2 assessment over different days depending on when researchers visited their schools and classrooms, this allowed variability in news scores. For instance, for participants who completed the T2 questionnaire on October 3^rd^, the composite scores were calculated as the total amount (sum) of news about ethnic minorities reported in the newspaper over the previous week (e.g., from September 26^th^ to October 2^nd^) divided by seven. Last, these scores were imputed into the main dataset using participants’ code, classroom, and school information to combine the newspaper and questionnaire data.

**Table S1**

Examples of coded news

| Valence by Target Code | News Extracted |
| --- | --- |
| Neutral News - Migrant | Today about one million of *immigrants* are living undocumented in the United Kingdom**.** |
| Positive News - Migrant | The truth is that not only is there no *immigration* emergency, but there are too few immigrants in Italy. |
| Negative News - Migrant | For Guetta, 'sovereignism' is the result of the confluence of populism and nationalism, fueling the re-legitimization of borders through protest in order to curb *migrants*, cultures, and products. |
| Neutral News - Refugee | To these children were added the children of *refugees* from Ukraine. |
| Positive News - Refugee | Biden offered 100 million dollars for hospitals, 200 million dollars for the UNRWA agency in charge of helping *refugees*, and other aid. |
| Negative News - Refugee | Or the question of Sweden and Finland joining NATO, with Turkey's objections due to the Kurdish *refugees* in the two Scandinavian countries, whose surrender Ankara, which considers them terrorists, demands. |
| Neutral News - Foreigner | Robert fights, with other *foreign* volunteers, alongside the regular Ukrainian army. |
| Positive News - Foreigner | In these conditions it is absurd to be afraid of *foreigners*. |
| Negative News - Foreigner | Often the feeling of spite for the problem being solved according to international rules, turns into offence at the fact that *foreigners* interfere. |

**Table S2**

Means (*M*) and standard deviation (*SD*) of study variables

|  | *M* | *SD* |
| --- | --- | --- |
| Affective prejudice T1 | 37.71 | 27.06 |
| Cognitive prejudice T1 | 1.78 | 0.73 |
| News Quantity | 2.73 | 1.42 |
| Neutral News | 1.58 | 0.96 |
| Positive News | 0.56 | 0.20 |
| Negative News | 0.59 | 0.27 |
| Neutral News – Migrants | 3.66 | 2.92 |
| Neutral News – Refugees | 0.27 | 0.24 |
| Neutral News - Foreigners | 0.83 | 0.26 |
| Positive News – Migrants | 1.28 | 0.66 |
| Positive News – Refugees | 0.09 | 0.12 |
| Positive News – Foreigners | 0.31 | 0.27 |
| Negative News – Migrants | 1.62 | 0.90 |
| Negative News – Refugees | 0.02 | 0.05 |
| Negative News - Foreigners | 0.14 | 0.13 |
| Affective prejudice T2 | 36.57 | 27.14 |
| Cognitive prejudice T2 | 1.93 | 0.75 |

*Note*. T = Time.

**Table S3**

Correlations among study variables

|  | 1. | 2. | 3. | 4. | 5. | 6. | 7. | 8. | 9. | 10. | 11. | 12. | 13. | 14. | 15. | 16. | 17. | 18. | 19. | 20. |
| --- | --- | --- | --- | --- | --- | --- | --- | --- | --- | --- | --- | --- | --- | --- | --- | --- | --- | --- | --- | --- |
| 1. Sex |  |  |  |  |  |  |  |  |  |  |  |  |  |  |  |  |  |  |  |  |
| 2. Age | -.02 |  |  |  |  |  |  |  |  |  |  |  |  |  |  |  |  |  |  |  |
| 3. Parents’ education | -.01 | -.10^**^ |  |  |  |  |  |  |  |  |  |  |  |  |  |  |  |  |  |  |
| 4. School track | -.08^*^ | .04 | -.36^***^ |  |  |  |  |  |  |  |  |  |  |  |  |  |  |  |  |  |
| 5. Affective Prejudice T1 | -.13^***^ | -.02 | -.11^**^ | .21^***^ |  |  |  |  |  |  |  |  |  |  |  |  |  |  |  |  |
| 6. Cognitive Prejudice T1 | -.16^***^ | .02 | -.09^*^ | .16^***^ | .49^***^ |  |  |  |  |  |  |  |  |  |  |  |  |  |  |  |
| 7. News Quantity | -.02 | .06 | .01 | .03 | .07 | .04 |  |  |  |  |  |  |  |  |  |  |  |  |  |  |
| 8. Neutral News | -.03 | .06 | .00 | .02 | .06 | .04 | .99^***^ |  |  |  |  |  |  |  |  |  |  |  |  |  |
| 9. Positive News | .01 | .05 | .07^*^ | -.03 | .06 | .02 | .97^***^ | .95^***^ |  |  |  |  |  |  |  |  |  |  |  |  |
| 10. Negative News | -.02 | .05 | .00 | .10^**^ | .08^*^ | .06 | .98^***^ | .96^***^ | .95^***^ |  |  |  |  |  |  |  |  |  |  |  |
| 11. Neutral News – Migrants | -.02 | .06 | .03 | .00 | .06 | .03 | .99^***^ | .99^***^ | .97^***^ | .96^***^ |  |  |  |  |  |  |  |  |  |  |
| 12. Neutral News – Refugees | -.03 | -.03 | -.12^***^ | -.02 | -.06 | -.02 | -.77^***^ | -.71^***^ | -.86^***^ | -.84^***^ | -.76^***^ |  |  |  |  |  |  |  |  |  |
| 13. Neutral News - Foreigners | -.05 | .03 | -.18^***^ | .33^***^ | .11^**^ | .14^***^ | .61^***^ | .60^***^ | .50^***^ | .67^***^ | .54^***^ | -.39^***^ |  |  |  |  |  |  |  |  |
| 14. Positive News – Migrants | .00 | .06 | .10^**^ | -.14^***^ | .02 | -.02 | .91^***^ | .91^***^ | .92^***^ | .84^***^ | .94^***^ | -.70^***^ | .23^***^ |  |  |  |  |  |  |  |
| 15. Positive News – Refugees | -.05 | .05 | -.08^*^ | .19^***^ | .10^**^ | .10^**^ | .93^***^ | .92^***^ | .85^***^ | .96^***^ | .90^***^ | -.69^***^ | .84^***^ | .70^***^ |  |  |  |  |  |  |
| 16. Positive News – Foreigners | .05 | -.05 | -.06 | .20^***^ | .02 | .04 | -.53^***^ | -.59^***^ | -.47^***^ | -.41^***^ | -.60^***^ | .14^***^ | .14^***^ | -.76^***^ | -.32^***^ |  |  |  |  |  |
| 17. Negative News – Migrants | -.03 | .05 | -.01 | .10^**^ | .08^*^ | .07 | .99^***^ | .97^***^ | .94^***^ | .99^***^ | .97^***^ | -.80^***^ | .68^***^ | .84^***^ | .97^***^ | -.45^***^ |  |  |  |  |
| 18. Negative News – Refugees | -.08^**^ | -.03 | -.13^***^ | .34^***^ | .05 | .12^***^ | -.11^***^ | -.13^***^ | -.25^***^ | .04 | -.15^***^ | .03 | .24^***^ | -.31^***^ | .14^***^ | .17^***^ | .04 |  |  |  |
| 19. Negative News - Foreigners | -.10^**^ | -.04 | .13^***^ | -.21^***^ | -.08^*^ | -.11^**^ | -.76^***^ | -.78^***^ | -.59^***^ | -.77^***^ | -.74^***^ | .35^***^ | -.67^***^ | -.58^***^ | -.85^***^ | .55^***^ | -.80^***^ | -.41^***^ |  |  |
| 20. Affective Prejudice T2 | -.18^***^ | .03 | -.17^***^ | .25^***^ | .73^***^ | .48^***^ | .04 | .03 | .01 | .05 | .02 | -.01 | .12^**^ | -.02 | .09^*^ | .03 | .06 | .12^**^ | -.10^**^ |  |
| 21. Cognitive Prejudice T2 | -.24^***^ | .05 | -.10^**^ | .12^**^ | .40^***^ | .67^***^ | .01 | .01 | -.03 | .03 | .00 | .04 | .13^***^ | -.06 | .08^*^ | .03 | .04 | .17^***^ | -.13^***^ | .52^***^ |

*Note*. 1. Sex: 0 = male, 1 = female. 3. Parents’ education was calculated as the sum of mother and father educational level, so that the higher the value, the higher the educational level of both parents combined. 4. School track: 0 = Academic-oriented track (i.e., lyceum), 1 = Technical and vocational tracks combined. T = Time.

^*^ *p* < .05; ^**^ *p* < .01; ^***^ *p* < .001

**S4. Longitudinal Measurement Invariance**

As a preliminary step, the longitudinal measurement invariance of both the affective prejudice and cognitive prejudice scales were tested separately. The configural models (for affective and cognitive prejudice) function as baseline models to attest measurement invariance and should therefore display a good fit, evaluated based on the following criteria. The Comparative Fit Index (CFI) with values higher than .90 and .95 indicates an acceptable and excellent fit, respectively. The Root Mean Square Error of Approximation (RMSEA) and the Standardized Root Mean Residual (SRMR) with values below .08 and .05 are indicative of an acceptable and very good fit, respectively (Byrne, 2012). Additionally, the RMSEA’s 90% confidence interval’s upper bound lower than .10 indicates an acceptable model fit (Chen et al., 2008). In order to establish metric invariance, changes in fit indices from the configural to the metric (i.e., a model where factor loadings are constrained to be equal across time) model were evaluated (e.g., Cheung & Rensvold, 2002). Specifically, a significant Δχ_SB_^2^ (Satorra & Bentler, 2001), and ΔCFI ≥ -.010 supplemented by ΔRMSEA ≥ .015 (Chen, 2007) are indicative of non-invariance. Metric invariance (which is the minimum requirement for regression analyses with observed variables) was established for both scales. Results are displayed in Table S4.

**Table S4**

Longitudinal Measurement Invariance of Prejudice Scales

| Models | Model fit | | | | |  |  | Model comparisons | | |
| --- | --- | --- | --- | --- | --- | --- | --- | --- | --- | --- |
|  | χ^2^ | df | CFI | SRMR | RMSEA  [90% CI] |  | Models | Δχ_SB_^2^ | ΔCFI | ΔRMSEA |
| Affective Prejudice | | | | | | | | | | |
| Configural (M1) | 180.818 | 47 | .965 | .033 | .055 [.047, .064] |  |  |  |  |  |
| Metric (M2) | 191.726 | 52 | .964 | .033 | .053 [.045, .062] |  | M2-M1 | 3.514 (5) | -.001 | -.002 |
| Cognitive Prejudice | | | | | | | | | | |
| Configural (M1) | 164.033 | 29 | .952 | .036 | .070 [.060, .081] |  |  |  |  |  |
| Metric (M2) | 165.746 | 33 | .953 | .039 | .065 [.055, .075] |  | M2-M1 | 4.934 (4) | .001 | -.005 |

*Note*. M = model; χ^2^ = chi-square; df = degree of freedom; CFI = Comparative Fit Index; TLI = Tucker-Lewis Index; SRMR = Standardized

Root Mean Square Residual; RMSEA = Root Mean Square Error of Approximation; CI = confidence interval; Δ = change in the parameter.

**S5. The moderating role of newspaper consumption**

To address the fourth and last aim of the present study, the main models were replicated within a multigroup framework and regression paths were compared to identify possible differences between adolescents with low and those with high levels of newspaper consumption. Results are detailed in Table S5 and further discussed in the manuscript. As can be inferred, adoelscents’ newspaper consumption did not significantly moderate any of the regression paths tested.

**Table S5**

Moderation analyses: Standardized regression coefficients across groups

|  | Low newspaper consumption  *β* (*SE*) | High newspaper consumption  *β* (*SE*) | Wald test^1^ |
| --- | --- | --- | --- |
| News quantity model | | | |
| Quantity → Affective prejudice | -.032 (.033) | .007 (.046) | 0.468 |
| Quantity → Cognitive prejudice | -.002 (.038) | -.055 (.057) | 0.574 |
| News valence model | | | |
| Neutral → Affective prejudice | .071 (.138) | .126 (.174) | 0.065 |
| Neutral → Cognitive prejudice | -.060 (.142) | .076 (.238) | 0.247 |
| Positive → Affective prejudice | -.152 (.147) | .040 (.164) | 0.761 |
| Positive → Cognitive prejudice | -.178 (.150) | -.427 (.210)^*^ | 0.777 |
| Negative → Affective prejudice | .046 (.149) | -.160 (.200) | 0.688 |
| Negative → Cognitive prejudice | .235 (.165) | .292 (.269) | 0.016 |
| Neutral news model | | |  |
| Migrants → Affective prejudice | -.030 (.057) | .042 (.084) | 0.507 |
| Migrants → Cognitive prejudice | -.064 (.061) | -.038 (.116) | 0.052 |
| Refugees → Affective prejudice | .046 (.051) | .043 (.066) | 0.001 |
| Refugees → Cognitive prejudice | -.006 (.052) | .052 (.089) | 0.307 |
| Foreigners → Affective prejudice | .052 (.047) | -.002 (.061) | 0.540 |
| Foreigners → Cognitive prejudice | .088 (.046) | .036 (.078) | 0.492 |
| Positive news model | | |  |
| Migrants → Affective prejudice | -.122 (.094) | .015 (.101) | 0.959 |
| Migrants → Cognitive prejudice | -.120 (.099) | -.263 (.130)^*^ | 0.657 |
| Refugees → Affective prejudice | .058 (.063) | -.009 (.067) | 0.522 |
| Refugees → Cognitive prejudice | .106 (.062) | .116 (.087) | 0.000 |
| Foreigners → Affective prejudice | -.043 (.062) | -.004 (.078) | 0.138 |
| Foreigners → Cognitive prejudice | -.010 (.065) | -.157 (.098) | 1.524 |
| Negative news model | | |  |
| Migrants → Affective prejudice | -.073 (.070) | -.038 (.088) | 0.089 |
| Migrants → Cognitive prejudice | .013 (.075) | -.120 (.110) | 0.965 |
| Refugees → Affective prejudice | -.003 (.043) | -.064 (.069) | 0.505 |
| Refugees → Cognitive prejudice | .044 (.048) | .082 (.092) | 0.052 |
| Foreigners → Affective prejudice | -.053 (.074) | -.050 (.092) | 0.001 |
| Foreigners → Cognitive prejudice | .010 (.075) | -.114 (.122) | 0.718 |

*Note*. *β* = standardized regression coefficient, *SE* = Standard error. ^1^All Wald test analyses had one

degree of freedom. All Wald tests were not statistically significant.

^*^ *p* < .05
